# First field documentation of interbreeding between released and wild *Callipogon relictus* (Coleoptera, Cerambycidae) signals in situ reinforcement progress in Korea

**DOI:** 10.3897/BDJ.14.e181698

**Published:** 2026-02-10

**Authors:** Jun-Young Kang, A Young Kim, Chang-Jun Kim

**Affiliations:** 1 Division of Forest Biodiversity, Korea National Arboretum, Pocheon, Republic of Korea Division of Forest Biodiversity, Korea National Arboretum Pocheon Republic of Korea

**Keywords:** conservation translocation, reinforcement, gene flow, radio telemetry, mating behaviour, captive breeding

## Abstract

Background

The relict longhorned beetle *Callipogon relictus* is protected in Korea as Natural Monument No. 218 and listed as an Endangered Species, Class I. Recent conservation measures include captive breeding and releases to bolster wild populations; however, empirical evidence of integration between released and wild individuals remains limited.

New Information

During daytime routine surveys in Gwangneung Forest, a radio-tagged female released on 10 July 2023 (LOTEK’s Pip4, model Ag337) was video-recorded copulating with an unmarked wild male on a *Quercus serrata* at 37°44′56.3″N, 127°09′04.3″E, at an elevation of 213 m. Copulation was first observed on 19 July and continued intermittently over 2 days, with repeated bouts lasting 10–20 minutes. This event occurred approximately 12 m aboveground on the leeward side of the *Q. serrata* trunk. The female radio-tracking tag was visible and did not appear to impede mating. Oviposition by the released female was later documented on a dead *Carpinus laxiflora* tree approximately 300 m away. This study constitutes the first formal field documentation of mating between released and wild *C. relictus*, indicating the onset of potential gene flow and meeting a key IUCN criterion for successful translocation (wild breeding).

## Introduction

The Korea National Arboretum (KNA) initiated a restoration programme for *Callipogon relictus* in 2016 and has secured a total of 34 wild-caught individuals from the Gwangneung Forest to date. To support captive propagation and conservation-orientated rearing, the KNA established the Forest Insect Smart Rearing Facility in 2020, under which the captive colony — derived from the Gwangneung Forest population — has been managed by periodically incorporating additional wild-caught individuals from the same population when available, as a precautionary measure to limit close-kin matings in captivity. Captive-reared individuals have been released in the Gwangneung Forest as part of the restoration and conservation programme since 2018 and radio tracking using attached radio tags has been conducted since 2021. Between 2021 and 2024, a total of 55 adults were released; radio tracking was successful for 36 individuals and carcasses were subsequently recovered for 23 of these individuals. During this monitoring, mating between a released female and a wild male followed by oviposition was documented only once (the observation reported here), whereas mating between released individuals was observed on two occasions. These observations provide a rare field context for evaluating whether released individuals can engage in wild breeding.

*C. relictus* is the largest Palaearctic cerambycid and a rare species found in East Asia. It is recognised as an endangered or extinct insect (Byun et al. 2007, Kuprin and Bezborodov 2012, Li et al. 2013, Bezborodov 2016, Yi et al. 2018). Its ecology in Gwangneung Forest and elsewhere has been clarified by long-term surveys and host-plant studies (Lee et al. 2018, Lee et al. 2019) and reinforcement via captive breeding and release has recently been implemented (Lee et al. 2021). However, empirical evidence for the integration of released and wild beetles remains undocumented.

Successful conservation translocations are ideally evaluated by post-release survival and breeding in the wild, together with demographic and genetic integration into recipient populations. For invertebrates, explicit field evidence of interbreeding between released and wild individuals is rare, although analogous success metrics exist (e.g. reintroduction of *Cerambyx cerdo* (Drag and Cizek 2015); reintroduction of *Nicrophorus americanus* (Saint Louis Zoo 2024).

Here, we report video-documented mating between a tagged released female and an unmarked wild male *C. relictus*. We interpret this as a critical milestone in situ reinforcement.

## Materials and Methods

**Study area and context**

Gwangneung Forest and adjacent areas, Republic of Korea comprise mixed deciduous forest with abundant mature host trees, where *C. relictus* adults are regularly recorded. Captive-reared individuals were released in 2023, following national permits. Host trees in Korea include *Carpinus laxiflora* and *Quercus* spp. (e.g. *Q. aliena* and *Q. serrata*), with the focal mating tree identified as *Q. serrata*.

**Daytime survey**

Routine daytime surveys were conducted daily from 09:00 h to 18:00 h during the adult activity period (late June to early September). Observers scanned from ground level to the treetops with unaided vision and binoculars and sightings were documented using a Nikon COOLPIX P950 (1920 × 1080, 30 fps). The released female carried a lightweight radio-tracking tag (LOTEK Pip4, Ag337; 0.31 g, a 20-day battery (13 × 5 × 3 mm)).

**Radio tracking and individual identification**

The LOTEK Pip4 (Ag337) tag was attached to the pronotum with a non-toxic adhesive to minimise interference with flight and identification numbers were applied to the elytra using marking fluid. The tracking used an ATS R410 receiver and an ATS 3-Element Folding Yagi antenna. Released females were verified from the release records. The male was unmarked and no released males were listed for this site/period, supporting a wild origin.

**Ethics (permits and welfare)**

Capture, temporary holding, tagging, release and tracking were conducted with permits from the Korea Heritage Service (Permission ID: Division of Natural Monument-5498 (2023-07-27)). Radio tags were attached to the pronotum using a non-toxic adhesive.

## Results

**Figure 1.**
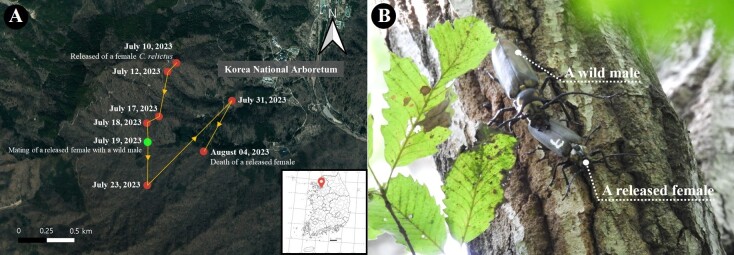
**A** The movement path of the released female *Callipogon relictus* individual; **B** Mating between the released female and a wild male of the same species.

On 10 July 2023, a captive-reared female *Callipogon relictus* was released near Sori-bong, Gwangneung Forest and monitored until 7 August 2023. Its movement path is shown in Fig. 1A. During post-release monitoring, the tagged released female was first recorded on 19 July, copulating with an unmarked wild male on a *Quercus serrata* (Fig. 1B). The pair remained together for two consecutive days, during which copulation occurred repeatedly through intermittent attachment and separation. Each copulation bout lasted 10–20 min and ended with natural separation; no investigator interference occurred. The location-tracking tag on the female dorsum was clearly visible and did not appear to impede the mating behaviour. Observations were made directly beneath the mating tree using 10 × 30 binoculars (ZEISS, Germany) and a digital camera (Nikon COOLPIX P950, 83× optical zoom); representative frames are shown in Fig. 1B and the full-field video is provided in Video S1. No other wild individuals were detected within the vicinity. The released female remained on the same tree for approximately 2 days, during which she mated, fed and subsequently departed. Oviposition by the released female was subsequently documented on a dead *Carpinus laxiflora* tree approximately 300 m away.

## Discussion

Captive breeding and subsequent release are widely used in conservation across taxa and can contribute to population restoration when carefully designed and evaluated (Armstrong and Seddon 2008, Sahashi and Morita 2022). Nonetheless, variable post-release performance, stemming from reduced wild adaptability or behavioural divergence, often constrains establishment (Robert 2009, Olzer et al. 2019). Accordingly, current reintroduction practices emphasise that released individuals survive, breed in the wild and ultimately contribute to recruitment as core success criteria (Griffith et al. 1989, Fischer and Lindenmayer 2000, Robert 2009).

In line with the IUCN/SSC Guidelines for Reintroductions and Other Conservation Translocations, translocation success depends on wild breeding, followed by recruitment (IUCN/SSC 2013). Our field documentation of mating between a tagged, released female and a wild male *C. relictus*, together with oviposition on a dead tree approximately 300 m away, meets the IUCN-defined milestone of wild breeding and signals the onset of gene flow towards demographic and genetic integration. We acknowledge, however, that, because the remaining Korean population is effectively restricted to a single regional population, reinforcement based on a limited founder base and multi-generation captive propagation may increase the risk of genetic bottlenecks and inbreeding if not carefully managed. Accordingly, our interpretation of “genetic integration” is provisional at this stage and requires confirmation through genetic monitoring of parentage and diversity across generations. To keep the IUCN aligned, we will continue standardised, non-invasive monitoring of behaviour and habitat use, genetic parentage and diversity, health status and mortality rates. These steps enable a criterion-based evaluation of translocation success under the IUCN framework and inform the adaptive management of the ongoing reinforcement programme.

## Conclusions

In this communication, we present the first formal field documentation of mating between a tagged released female and a wild male *C. relictus*, indicating the onset of potential gene flow and meeting a key IUCN criterion for translocation success (wild breeding). Follow-up monitoring further documented oviposition at a separate site approximately 300 m from the mating tree.

## Data Resources

The original field video (Video S1, Full HD 1920 × 1080, 30 fps) was archived at Zenodo (https://doi.org/10.5281/zenodo.17230325). The additional field video (Video S2, 2320 × 1080, 29.99 fps, 10 s; audio: 48 kHz, stereo) was archived at Zenodo (https://doi.org/10.5281/zenodo.18218167).
